# Advancement in modeling of Alzheimer’s disease: a comprehensive review of preclinical screening platforms

**DOI:** 10.3389/fnagi.2025.1646551

**Published:** 2025-08-06

**Authors:** Souvik Adak, Shivam Singh, Ridhi Jain, Abhilasha Tiwari, Binny Singh, Sumit Kumar, Ayushi Dadhwal, Avishek Chakroborty, Ashish Kumar Sharma, Ravindra Pal Singh, Rajesh Kumar Sharma

**Affiliations:** NIMS Institute of Pharmacy, NIMS University, Jaipur, India

**Keywords:** Alzheimer’s disease, cognitive impairment, APP, tau, neurofibrillary tangles, screening models

## Abstract

Alzheimer’s disease (AD) is a chronic and progressive neurodegenerative condition that worsens with time and causes memory loss and cognitive impairment. For prompt intervention and management of AD, early detection is essential. Screening models play a crucial role in identifying individuals at risk of developing AD before the onset of noticeable clinical symptoms. This review summarizes a wide range of *in vitro* and *in vivo* screening models currently utilized in AD research, highlighting their advantages and limitations. *In vitro* systems-such as cell lines and primary neuronal cultures-provide controlled settings to investigate cellular mechanisms and drug efficacy. In contrast, *in vivo* models, including transgenic rodents and other animals, better replicate the complex biological features of AD. Each model type comes with distinct benefits and limitations concerning clinical relevance, cost-effectiveness, and ethical challenges. By evaluating the utility and constrains of these models, this article seeks to assist researchers in choosing suitable platforms for preclinical investigations and support the advancement of improved diagnostic tools and therapeutic strategies for AD.

## 1 Introduction

Alzheimer’s disease (AD) is a progressive neurodegenerative disorder that initially presents with subtle memory impairments and difficulty in performing routine tasks. As the condition advances, it leads to significant cognitive dysfunction, behavioral changes, and a decline in functional abilities, ultimately compromising the individual’s independence and quality of life (Furiak et al., 2010; Li et al., 2016). AD is the leading cause of dementia in the elderly population, accounting for approximately 60%–70% of all dementia cases. It affects millions globally and imposes a substantial burden on patients, caregivers, and healthcare systems. In 2023, it was estimated that around 6.7 million Americans aged 65 and older were living with Alzheimer’s dementia. This number is expected to nearly double by 2060, reaching approximately 13.8 million, assuming no major advances in treatment. AD affects roughly 11% of individuals over 65, and its prevalence rises with age (Alzheimer’s Dementia, 2023). Since its initial description by Dr. Alois Alzheimer in 1906, considerable progress has been made in understanding AD. However, many aspects regarding its etiology, progression, and management remain unresolved (Blaikie et al., 2022; Schaeffer et al., 2011; Shaughnessy et al., 2004). Pathologically, AD is marked by widespread synaptic and neuronal loss, extracellular amyloid-beta (Aβ) plaque accumulation, and intracellular neurofibrillary tangles composed of hyperphosphorylated tau protein. These pathological changes are especially prominent in brain regions associated with memory and cognition, such as the hippocampus and cerebral cortex. A significant feature of AD is the degeneration of cholinergic neurons in the basal ganglia forebrain, which innervate key regions including the neocortex, hippocampus, and amygdala, leading to impaired cholinergic transmission (Guénette and Tanzi, 1999; Janus et al., 2004).

The AD symptoms correlate with amyloid deposition and neurodegeneration, which initially affect the hippocampus and cortical regions and eventually extended to the cerebellum and brainstem as the disease progresses (Janus et al., 2004; Woodruff-Pak, 2008). While age remains the most significant risk factor, genetic, environmental, and life style components also contribute. For instance, the apolipoprotein (APOE4) allele has been strongly associated with increased with increase susceptibility to AD. Additionally factors such as cardiovascular health and social management have been implicated in modulating disease (Lee and Han, 2013; Trinchese et al., 2004; Wenhai et al., 2016). Despite decades of intensive research, translating molecular insights into effective treatment has been challenging. A key obstacle is the lack of fully representative models that recapitulate the complex pathophysiology of human AD (Lee and Han, 2013). This review aims to provide a comprehensive overview of currently available models for AD and evaluate their effectiveness in elucidating disease mechanisms and testing therapeutic interventions. While no model can fully mimic the human conditions, animal models remain indispensable for bridging the gap between molecular research and clinical application. Valid non-human models are essential for exploring pathogenic pathways, assessing drug efficacy, and evaluating preventive strategies, and identifying potential biomarkers relevant to different stages of AD (Myers and McGonigle, 2019; Woodruff-Pak, 2008).

To understand how the experimental models translate molecular pathology into functional impairment, it is critical to examine the behavioral phenotypes associated with AD. Behavioral assays serve as essential tools in preclinical studies by offering quantifiable measures of cognitive deficits that mirror clinical symptoms. These tests provide insight into memory, learning, anxiety, and spatial navigation-all of which are disrupted in AD. In this article, we also outline step-by-step behavioral protocols commonly used in rodent models of AD, discussing their relevance, methodological approaches, and interpretive value in assessing disease progression.

## 2 Screening models for AD

### 2.1 Behavioral paradigms for evaluating memory and learning

#### 2.1.1 Passive avoidance tests

##### 2.1.1.1 Step down model

**Principle:** An animal (rodent) will spend a large portion of its time in the corners and close to the walls of an open field. When positioned on a raised platform in the center of a rectangular compartment, it almost immediately descends to the floor to explore the enclosure and approaches the wall. This initial step-down latency, along with the response to subsequent stimuli such as mild foot shock or aversive cues, is used to evaluate memory retention and learning ability in response to pharmacological or genetic interventions (Gao et al., 2015; Kurup et al., 2020).

**FIGURE 1:**
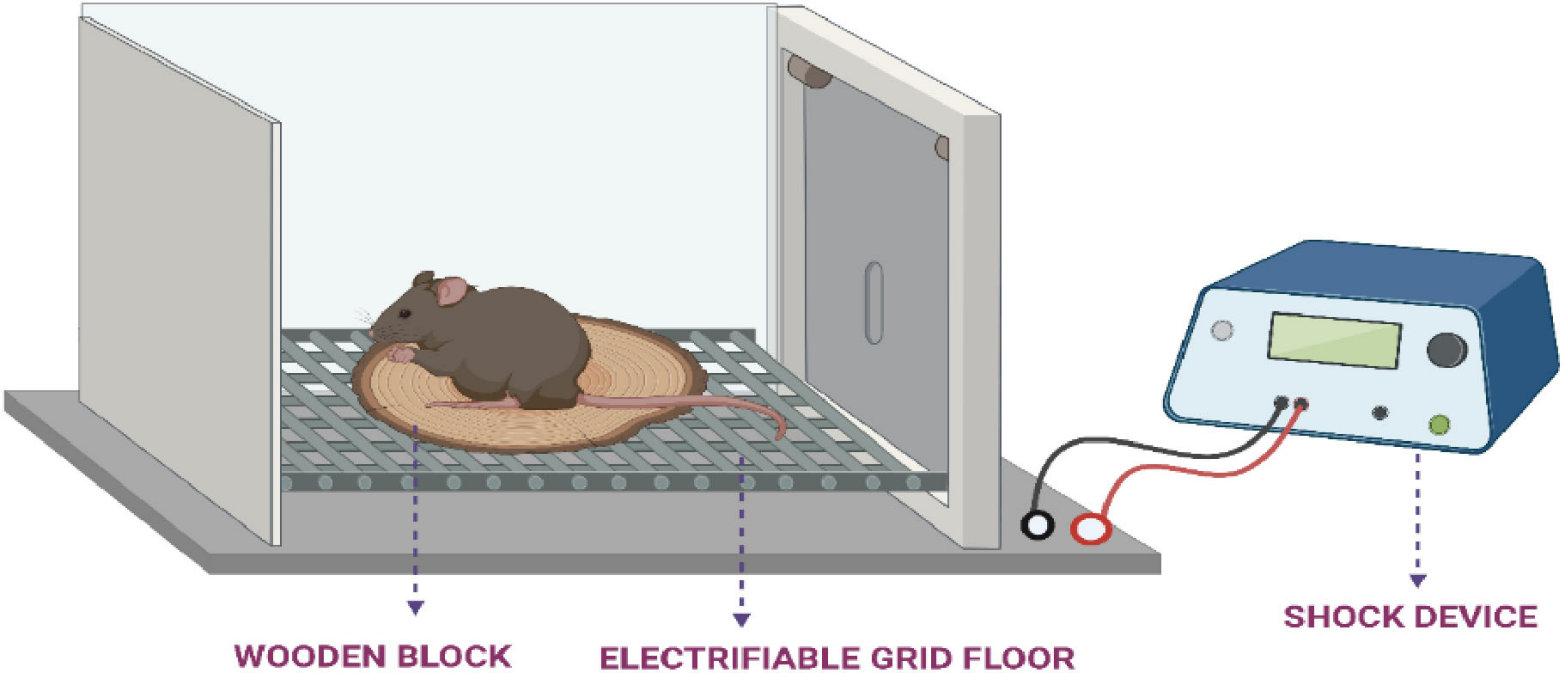
Experimental setup of the step-down passive avoidance test, used to assess memory retention in rodents.

**Methodology:** In this experimental setup (Figure 1), commonly used subjects are laboratory rodents such as mice or rats, chosen for their suitability in behavioral research. To induce cognitive impairment, an amnestic agent like phenytoin may be administered. The apparatus typically includes a square chamber (around 50 cm per side) featuring a metal grid floor capable of delivering mild foot shocks, along with a small elevated platform placed at the center, approximately 3.5 cm above the floor level (Kurup et al., 2020). The procedure starts with dividing the animals into two groups: a control group and a test group. The procedure is conducted in three stages. In the first stage, known as the familiarization phase, the animal is placed on the raised platform after the removal of a surrounding cylinder. The latency to descend from the platform is recorded, after which the animal is allowed to explore the enclosure for 10 s before being returned to its home cage. In the second stage, referred to as the learning phase, the animal is again placed on the platform. Upon descending, it receives an unavoidable mild foot shock (50Hz, 1.5 mA for 1 s), which serves as an aversive stimulus. The animal is then promptly returned to its home cage. The third stage, the retention test, is conducted 24 h after the learning phase. The animal is placed once again on the platform, and the step-down latency is measured. The trial ends when the animal descends or after a maximum of 60 s, which is used as the cut-off time. To evaluate memory impairment, a known amnestic agent, such as phenytoin, is administered to the test group animals 24 h after the retention test. The behavioral test is then repeated, and any reduction in step-down latency compared to the control group is interpreted as an indication of impaired memory performance (Gao et al., 2016; Shen et al., 2020; Zhang et al., 2011; Zhang et al., 2016).

A wide range of studies had employed this passive avoidance test as a behavioral tool to assess learning and memory impairments in experimental models of AD. For instance, a study examined the impact of pioglitazone on cognitive impairment induced by scopolamine in mice. Scopolamine caused a notable decline in step-down latency, reflecting memory dysfunction. Treatment with pioglitazone resulted in a dose-dependent improvement in latency, indicating its ability to counteract memory deficits (Gupta and Gupta, 2012). Further, Pinton et al. (2013) employed step-down model to investigate the therapeutic potential of organoselenium dietary supplementation in a streptozotocin (STZ)-induced model of AD. Results from step-down passive avoidance test showed a reduced memory deficits linked to the condition (Pinton et al., 2013), highlighting the role of this model in AD research.

The step-down model offers a straightforward and economical approach to evaluate memory impairments, making it valuable for initial testing of potential cognitive enhancers (See Table 1). Nonetheless, its focus on short-term, fear related memory, lack of relevance to the full complexity of AD pathology, and susceptibility to stress-induced variations limit its overall applicability (Feng et al., 2004; Gupta and Gupta, 2012).

**TABLE 1:** Comparative evaluation of traditional and newer behavioral models used in AD research.

Parameters	Conventional models (e.g., MWM, Y-maze, passive avoidance)	Step-down/step-through models
Stress involved	Often high; tasks like water maze may induce anxiety or physical stress	Minimal stress; no swimming or intense physical effort
Ease of training and setup	Time-intensive; may require several days of habituation and trails	Quick to administer; training is simple and brief
Dependency on motor function	High; tasks require good coordination and mobility	Low; minimal movement reduces motor related confounding
Detection of mild cognitive impairment	May overlook subtle deficits, especially in early stages	High sensitivity to changes in learning and memory
Memory type assessed	Broad; includes spatial, working, and avoidance memory	Primarily assesses fear-associated (aversive) memory
Effect of repeated testing	Typically, consistent over trials	May show reduced response due to habituation
Influence over handling	Moderate; varies with animal temperament and environment	Can be more sensitive to inconsistencies in handling
Suitability for aged or impaired models	Challenging; motor deficits can impact outcomes	More appropriate; low physical demands
Breadth of cognitive domains covered	Wide range including spatial learning and decision-making	Narrow focus; limited to avoidance-based memory

##### 2.1.1.2 Step through method

**Principle:** The step-through passive avoidance test utilizes the natural behavior of rodents, such as mice and rats, which prefer dimly lit or dark environments and tend to avoid brightly illuminated areas. When placed in a well-lit compartment connected to a dark chamber, the animal instinctively enters the darker side and remains there. This behavioral tendency is used to study learning and memory by associating the dark chamber with an unpleasant stimulus. The test was initially standardized for mice by Jarvik and Kopp in 1967, with subsequent modifications made by King and Glaser in 1970 to adapt the protocol for use in rats (Feng et al., 2004; Rabiei et al., 2013).

**FIGURE 2:**
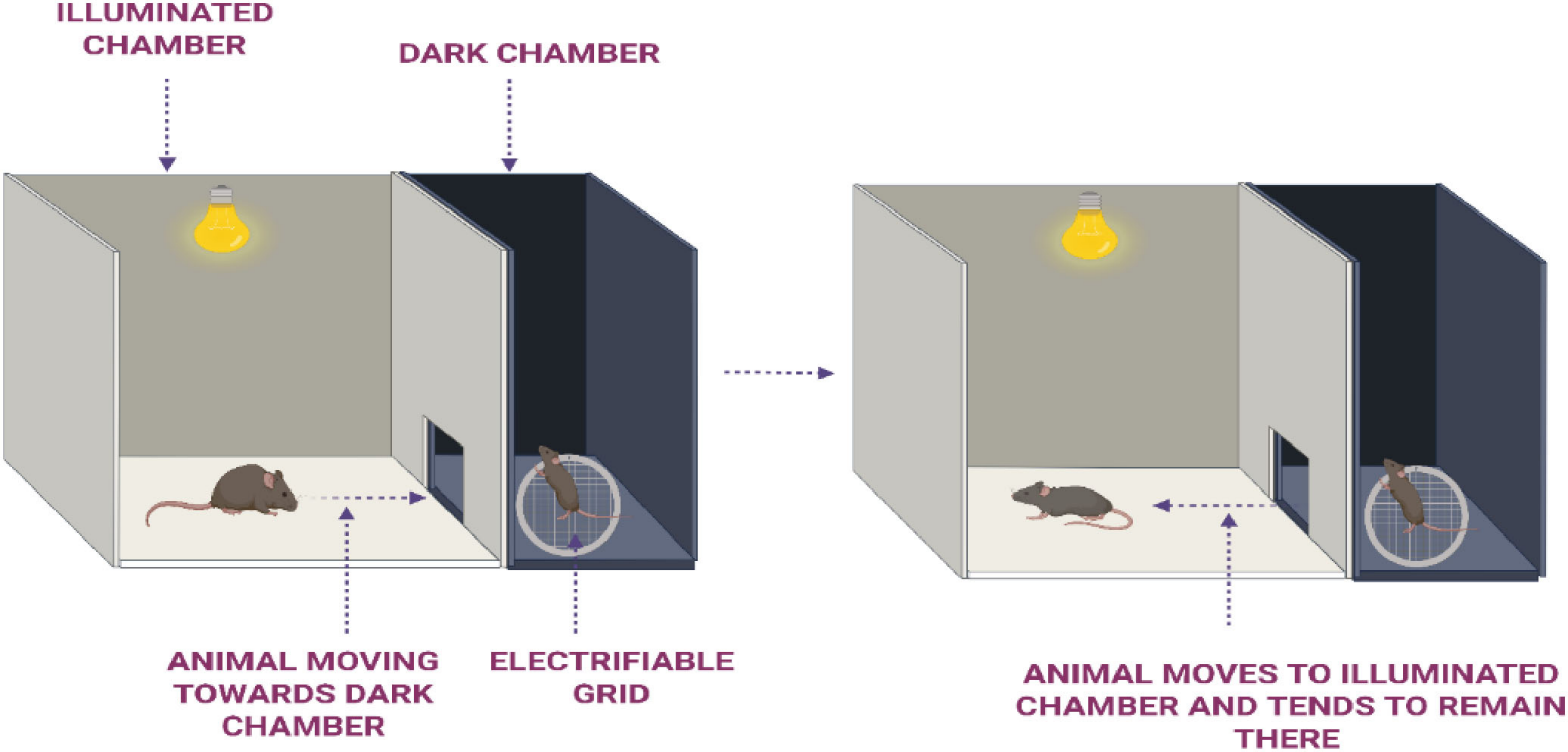
Step-through passive avoidance test to assess memory retention in rodents.

**Procedure:** The apparatus (Figure 2) used in this test consists of a small illuminated compartment connected to a larger dark chamber via guillotine door. A 7W/12V light bulb is used to illuminate the smaller compartment, leveraging the rodent’s natural aversion to light and preference for dark environments (Feng et al., 2004; Jung et al., 2014).

The experiment is conducted using two groups: a control group and a test group. During the acquisition trial, the animal is placed in the illuminated chamber, positioned as far from the guillotine door as possible. The latency to enter the dark compartment is recorded. If the animal fails to enter the dark chamber within a predefined time limit-90 s for mice and 180 s for rats-it is excluded from further testing. Upon entry into the dark chamber, the door closes automatically, and the animal receives an unavoidable mild foot shock (1 s at 1.0 mA for mice; 2 s at 1.5 mA for rats). The animal is then promptly removed from the apparatus (within 10 s) and returned to its home cage. A retention trial is conducted 24 h after the acquisition phase, during which the animal is again placed in the illuminated chamber. The latency to re-enter the dark compartment is measured, with an extended cut-off time of 300 s for mice and 600 s for rats. Increased latency during the retention test is considered indicative of memory retention. Following this phase, a known amnestic agent (example: Scopolamine) is administered exclusively to the test group, and the procedure is repeated to evaluate the impact of the compound on memory performance (Nikseresht et al., 2021; Nobakht et al., 2011).

A recent study investigated the effect of cacao consumption on cognitive deficits in an AD rat model. The study demonstrated that cacao administration significantly improved memory retention, as evidenced by increased latency in the step-through passive avoidance test (Basir et al., 2024). Similarly, another study employed this model to examine the therapeutic effect of Selegiline on cognitive function in AD rats. Selegiline-treated animals exhibited significantly increased latency to enter the dark compartment during retention trials, indicating improved memory performance (Mohamadpour et al., 2024). These findings validate the significant role of step-through model in evaluating behavioral changes associated to AD. This is a simple and cost-effective approach for AD studies. Despite its utility, the model is limited by stress-related factors, potential interference from non-memory influences, and its narrow focus, which may not capture the full complexity of AD-related cognitive decline (Jung et al., 2014).

#### 2.1.2 Active avoidance test

##### 2.1.2.1 Runway avoidance

**Principle:** The runway active avoidance test operates on the concept of learned behavior through negative reinforcement. In this paradigm, an animal is placed in a straight corridor or runway where it is trained to escape an impending aversive stimulus-typically a mild foot shock-by moving to a designated safe zone. A sensory cue (such as a light or tone) serves as a warning signal preceding the shock. If the animal successfully reaches the safe zone within a predetermined time, the aversive stimulus is avoided. With repeated trials, the animal learns to associate the warning cue with the need to escape, reflecting its ability to acquire and retain information through experience. This test is particularly valuable for assessing learning efficiency, cognitive flexibility, and the effects of pharmacological agents on avoidance behavior (Utkan et al., 2012).

**FIGURE 3:**
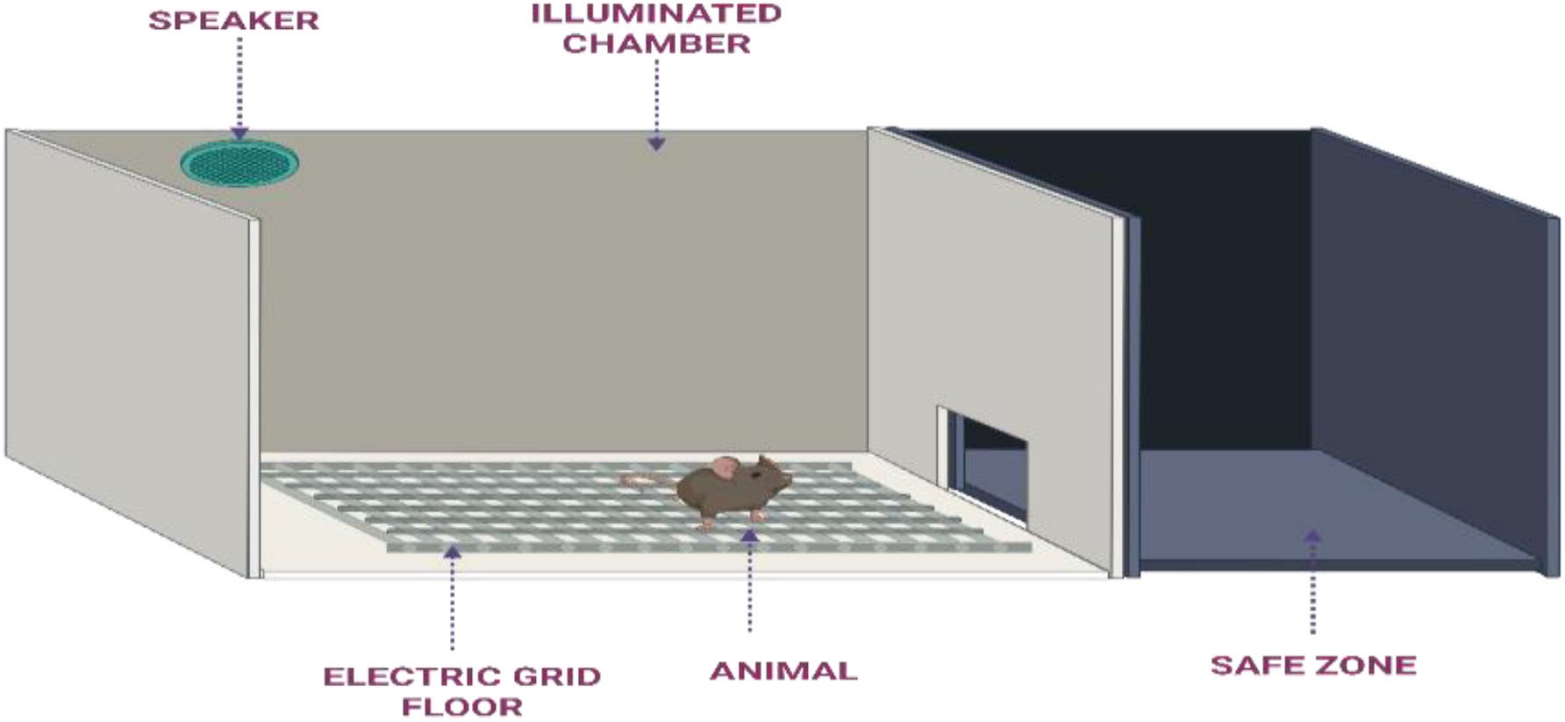
Illustration of the step-through active runway-avoidance apparatus used for memory assessment in rodent models.

**Methodology:** This model assesses learning and memory in rodents using a modified apparatus (Figure 3) similar to that used in the step-through avoidance test, with the addition of a loudspeaker to deliver an auditory conditioned stimulus (CS). Male or female mice or rats are housed under standard laboratory conditions and acclimatized through daily handling before the experiment begins. An overhead light source ensures consistent illumination within the apparatus, while a speaker is positioned approximately 50 cm above the starting compartment to emit the auditory cue (Setogawa et al., 2014). Initially, the animal is allowed to freely explore the apparatus for 5 min. After this habituation period, the guillotine door is closed and the animal is placed in the illuminated compartment. Following a 10 s interval, the door opens and the auditory stimulus begins. 5 s later, a mild foot shock is delivered via the floor grid. The auditory stimulus continues until the animal reaches the designated safe area, reinforcing the association between the CS and the need to avoid the aversive stimulus. Once in the safe zone, the animal remains there for an intertrial interval of 50–70 s before being repositioned for the next trial. After a 30 s delay, a mechanical prompt forces the animal back onto the grid floor, initiating the next trial. This cycle is repeated until the animal achieves ten consecutive successful avoidance responses. A retention test is conducted 24 h later, where the same procedure is repeated to evaluate memory consolidation and recall. To assess memory deficits, a known impairing agent is administered to the test group prior to the retention trial. The performance of each animal is evaluated based on the time taken to reach the safe zone (latency) and the number of errors made (failures to avoid the shock). These parameters are compared between control and treated animals to determine the impact of the intervention on learning and memory processes (Mutlu et al., 2011; Ohno et al., 1990).

This model had also been exclusively utilized in AD researches. Setogawa et al. (2014) used the runway avoidance model to evaluate memory-guided locomotion in Ad, employing triple transgenic mice. The model revealed that these mice made more hindlimb contacts with obstacles than controls, indicating deficits in cognitive-motor coordination likely linked to impaired memory. This highlights the runway avoidance model as a useful tool for assessing cognitive-motor integration in AD (Setogawa et al., 2014).

Despite its usefulness, the test can be affected by the animal’s stress levels, animal’s motivation, and learning ability. Moreover, it doesn’t isolate specific cognitive functions, which can limit its interpretive clarity (Setogawa et al., 2014; Utkan et al., 2012).

##### 2.1.2.2 Jumping avoidance (one-way shuttle box)

**Principle:** The jumping avoidance model in a one-way shuttle box introduces a vertical component to enhance the distinction between the starting and goal areas. Unlike standard horizontal movement tasks, this model requires the animal to perform a distinct, all or none motor response-specifically, a jump-to avoid an aversive stimulus. This vertical response adds complexity to the task, encouraging a more defined behavioral outcome that contrasts with the gradual or continuous movements typically observed in conventional avoidance paradigms. By demanding a clearly delineated escape behavior, the model allows researchers to assess discrete motor learning and decision-making processes under aversive conditions (Cheng et al., 2014).

**Methodology:** This model employs rodents (rats preferred) of both sexes and uses a specialized rectangular apparatus measuring 40 × 25 cm, enclosed by metal walls rising 40 cm high and covered by a Plexiglas ceiling. The floor consists of an electrified grid to deliver foot shocks. A transparent plastic pedestal (12 × 12 × 25 cm), attached to one of the short walls of the apparatus, serves as the goal area. A vertically movable barrier is positioned flush with the pedestal’s horizontal surface. This barrier can either obstruct the animal’s access to the goal or retract to reveal the safe zone against the back wall of the chamber (Walker et al., 2015).

Initially, each animal is allowed to acclimate in the apparatus for 5 min. The goal platform is then briefly revealed by retracting the barrier, following by closing the barrier for 2 s. Subsequently, the goal area is exposed again, and an auditory CS of 1000 Hz at 85 dB is presented. 5 s later, or at intervals of 2 s, an unconditioned stimulus (US) in the form of a foot shock (0.5 s, 1.0 mA at 50 Hz) is applied. This pairing of CS and US continues until the animal performs a discrete avoidance response-jumping onto the pedestal. After a 3 min rest, the barrier displaces the animal from the platform, placing it back on the grid floor. This cycle is repeated until the animal meets the learning criterion of ten consecutive successful avoidances. Retention is assessed 24 h later under the same conditions, where the animal is again required to reach the platform with similar performance consistency. To determine the memory impairment an amnestic agent is administered to the test group 30–60 min prior to the retention test (Flicker et al., 1983; Stepanichev et al., 2005). The primary evaluation parameters include the latency to reach the goal platform and number of errors, defined as failures to avoid the latency to reach the goal platform and the number of errors, defined as failures to avoid the shock by not reaching the safe area in time. Comparison of these parameters between training and retention sessions helps determine the animal’s learning and memory performance (Middei et al., 2004).

Compared to other behavioral models, the jumping avoidance test is used less frequently in AD research. Although it can effectively assess certain forms of memory, such as fear-associated and long-term memory, it doesn’t adequately capture broader cognitive impairments like spatial or working memory, which are commonly affected in AD. Its reliance on aversive stimuli may introduce stress-related variables, and differences in how animal strains respond to such stress further limit its consistency. These factors contribute to its limited application, promoting researchers to favor more comprehensive and validated models (Cimadevilla et al., 2000; Middei et al., 2004).

#### 2.1.3 Discrimination learning

##### 2.1.3.1 Open field test (OFT)

**Principle:** The OFT is based on a rodent’s instinctive tendency to explore unfamiliar environments, driven by their search for resources such as food, shelter, or mating partners. When introduced to a novel arena, animals typically show heightened exploratory behavior. Upon repeated exposure to the same environment, this behavior gradually decreases, reflecting a process known as spatial habituation. This reduction is interpreted as the animal’s ability to recognize and remember the spatial features of the setting. In the context of AD research, a failure to exhibit normal habituation may indicate impairments in recognition memory and spatial learning. Therefore, the OFT serves as a useful tool for identifying early cognitive deficits and assessing the efficacy of potential therapeutic agents in experimental AD models (Ribes et al., 2008; Whyte et al., 2018).

**FIGURE 4:**
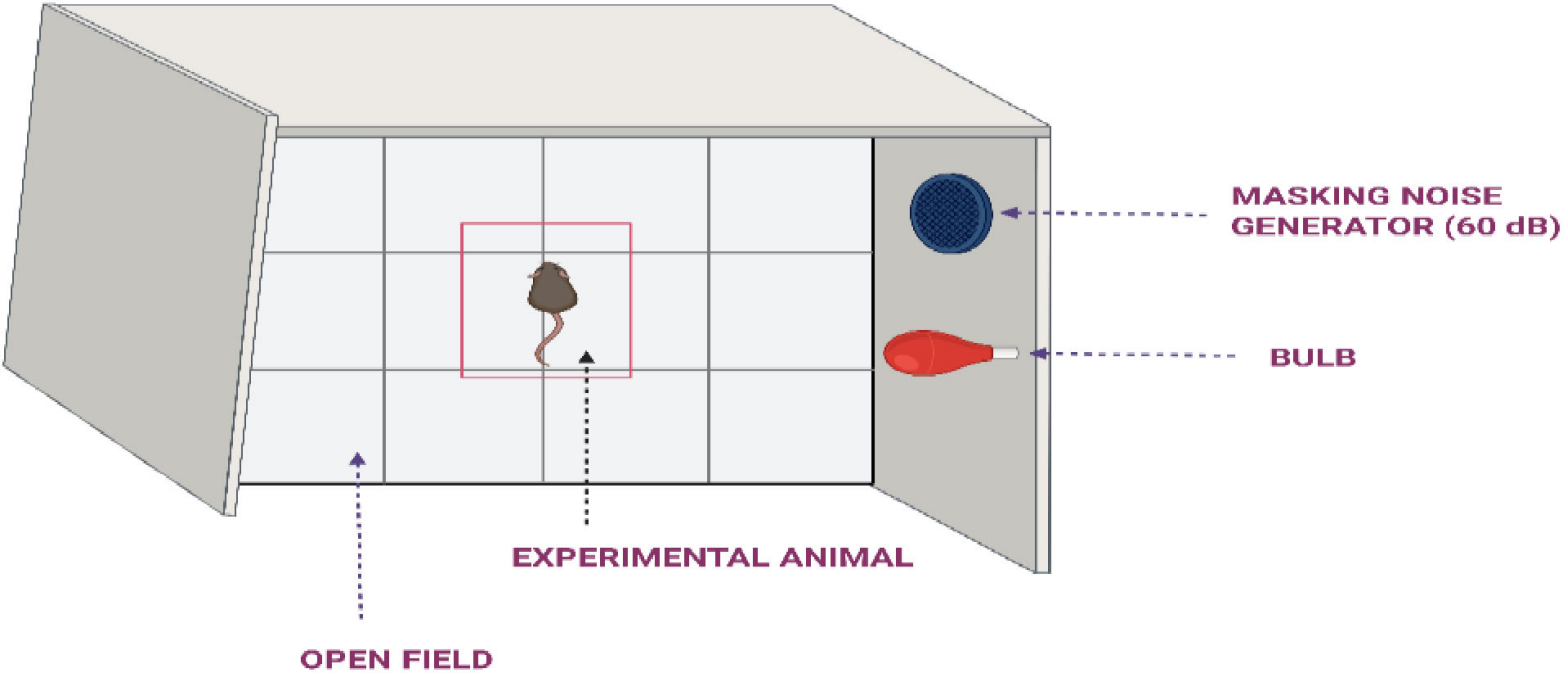
Representation of the open field discrimination learning task used in cognitive behavioral assessment.

**Methodology:** The open field apparatus (Figure 4) is a rectangular chamber made of gray PVC or painted wood, with dimensions designed to allow sufficient space for the animal’s movement. A 25 W green or red lightbulb is positioned either above or below the maze to provide uniform illumination of around 0.3 1x in the center. In addition, a wide-spectrum noise source generating 60 dB of sound is used to produce masking noise, minimizing environmental distractions during testing. The chamber is thoroughly cleaned with alcohol before each trial to eliminate odors from prior experiments. Before testing, animals are allowed to acclimate in the assessment room for 30 min to reduce stress. The quarantine area and testing room are kept separate to avoid any potential cross-contamination of scents. During the experiment, the animal’s trajectories are continuously monitored and recorded. Testing typically occurs during the dark phase of the animal’s circadian cycle, especially for older or hypoactive rodents. The rodent is placed either in a corner or in the center of the open filed for a testing duration of 5–10 min (up to 20 min for more active animals such as mice. After the initial exposure, the animals are re-exposed to the open field 24 and 96 h later to evaluate changes in behavior and memory consolidation (García et al., 2009; Pentkowski et al., 2021; Sterniczuk et al., 2010). To evaluate the memory deficit amnestic agent such as phenytoin or scopolamine is administered to the animals of test group approximately 30–60 min before the re-exposure trials at either 24 or 96 h post initial training. The timing is selected based on the pharmacological profile of compound and allows for the assessment of its impact on exploratory behavior and habituation, reflecting deficits in cognitive processing (Kraeuter et al., 2019).

Horizontal activity, represented by the total distance moved within the open field, is used to assess overall locomotor function. Vertical activity, observed as rearing behavior, reflects the animal’s exploratory tendencies and environmental engagement. The duration of rearing may also indicate levels of non-specific attentiveness. Additional measures, such as the time spent in central versus peripheral zones, frequency of grooming, and the number of fecal droppings, are commonly used as indirect markers of anxiety-related behavior which are often used as indicators of cognitive function and emotional state in rodents (Filali et al., 2012; Lim et al., 2001).

Compared to other models, this model is more commonly employed in AD researches. Hashiguchi et al. (2020) explored how resistance exercise influenced behavior in an AD mouse model. They observed that exercise reduced the increased locomotor activity exhibited by these mice in open field test, suggesting an improvement in motor behavior associated with AD (Hashiguchi et al., 2020). Similarly, in a 2018 study, the open field test was used to measure spontaneous movement and exploration in the AppNL-G-F knock-in mouse model of AD. The mice demonstrated decreased locomotor activity and exploration compared to controls, despite no noticeable memory problems at that time (Whyte et al., 2018). This suggests that the open field test can effectively detect early motor and extrapolatory impairments in AD models before cognitive symptoms become apparent.

The open field test mainly evaluates locomotor activity and anxiety-related behavior; however, its results can be affected by various non-cognitive influences such as motor impairments, stress reactivity, or age-related decline, limiting its specificity for assessing cognitive dysfunction. Repeated exposure to the test environment may cause habituation, diminishing its effectiveness in tracking disease progression or therapeutic outcomes (Hashiguchi et al., 2020; Walf and Frye, 2007).

##### 2.1.3.2 Y-maze

**Principle:** The Y-maze is used to assess spatial working memory by encouraging the animal to differentiate between arms-one that is illuminated and safe, and another that is dark and associated with an aversive stimulus. The animal learns to consistently choose the illuminated arm, indicating its ability to retain and apply spatial information (Kraeuter et al., 2019).

**FIGURE 5:**
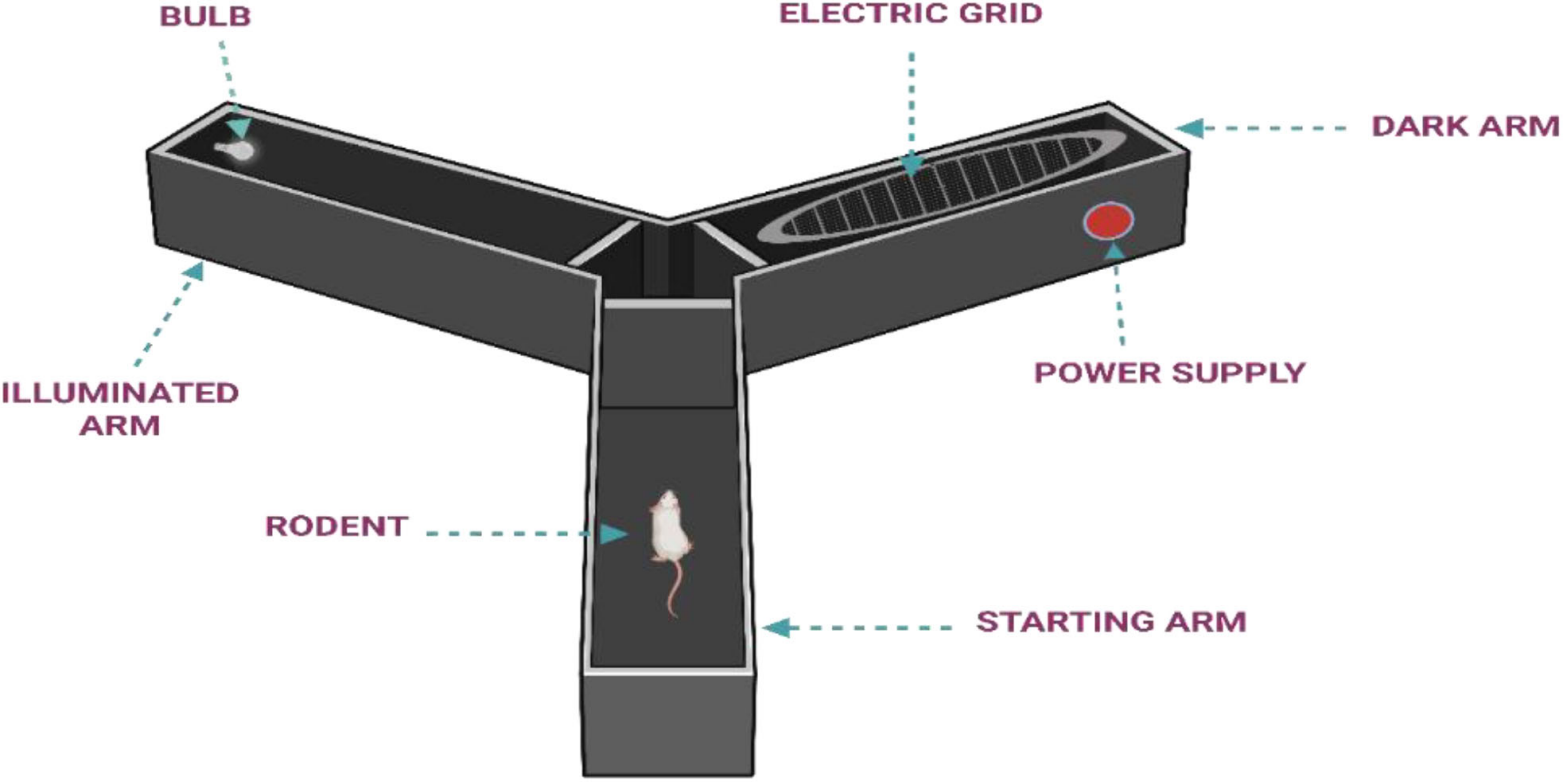
Illustration of Y-maze apparatus.

**Procedure:** To begin the experiment, animals are given a 5 min period to explore the Y-maze apparatus (Figure 5) freely, allowing them to acclimate to the environment. After this habituation phase, each animal is placed in the start chamber. A mild aversive stimulus is applied-either through activation of a shock generator or by selecting the appropriate program-once the animal begins moving toward the illuminated arm, typically within 5 s. Upon entering the lit arm, the animal is allowed to remain there for a predetermined intertrial interval before the next trial begins. This process is repeated using the pre-defined sequence in the program. Training is continued until the animal demonstrates consistent learning, usually defined as making nine correct choices out of ten consecutive trials. To assess memory impairment, an amnestic agent is administered to test grouped animals prior to the final trial or retention test, depending on the experimental design. This allows for evaluation of how the agent affects previously acquired learning or working memory (Ognibene et al., 2005; Stewart et al., 2011). Behavioral assessment focuses on the total number of arm entries and animal’s alteration behavior, which serves as an indicator of working memory performance. A high percentage of arm entries and the animal’s alteration behavior, which serves as an indicator of working memory performance. A high percentage of correct choices, particularly 9 out of 10 accurate entries, is indicative of effective learning and intact memory function (Nobakht et al., 2011; Stover et al., 2015).

Y-maze offers several advantages, including simplicity, low cost, minimal stress to animals, and sensitivity to hippocampal dysfunction. However, it has limitations such as assessing only short-term memory, being influenced by changes in locomotor activity, and lacking insight into the broader cognitive and neurobiological aspects of AD. Therefore, while useful for initial screening, it should be complemented with other behavioral and molecular assessments for a more comprehensive evaluation (Kraeuter et al., 2019; Vorhees and Williams, 2014).

##### 2.1.3.3 Morris water maze (MWM)

**Principle:** The MWM operates on the concept of spatial learning and memory, where rodents must locate a hidden platform (HP) in a circular pool of opaque water using external visual cues around the testing area. Through repeated trials, the animal learns the platform’s location, and its ability to recall this position-especially during probe trials when the platform is removed- serves as a measure of cognitive performance related to the hippocampus, making it a key tool in AD research (Vorhees and Williams, 2006).

**Methodology:** The setup consists of a large circular pool, approximately 2 m in diameter, filled with opaque water maintained at 25–27°C. The water’s opacity is achieved by adding a non-toxic white dye or suspension agent to obscure a submerged escape platform located about 1 cm beneath the surface in one of the four quadrants. A heating system ensures a stable temperature throughout the trials (Othman et al., 2022; Vorhees and Williams, 2006). External visual cues are fixed around the pool to serve as spatial references (Vorhees and Williams, 2006). An overhead camera records the trials, and behavioral tracking software is used to analyze metrics such as escape latency, path length, and swim speed (Garthe et al., 2009). Animals are acclimated to the testing environment before beginning the protocol. The training phase is typically divided into two stages: visible platform (VP) training and HP training. During the VP phase, the platform is marked with a visible object to ensure the animal can associate swimming with an escape route. Its position is varied across sessions to prevent reliance on positional memory. Mice are released from randomized start positions and are allowed a maximum of 60 s per trial. If they fail to find the platform, they are gently guided to it and permitted to remain there for 30 s. This training continues for 4 days, with multiple trials per day. Mice unable to locate the platform consistently during VP training may be excluded from further testing (Othman et al., 2022; Vorhees and Williams, 2006). In the HP phase, the visible cue is removed, and the platform remains in a fixed location. Animals are released from different start points in a semi-randomized sequence across several days of testing. Performance is evaluated over four trials daily, with each separated by approximately 20 min. Probe trials are conducted periodically (e.g., on days 4, 7, and 10) by removing the platform and allowing the animal to swim freely for 60 s (D’Hooge and De Deyn, 2001; Vorhees and Williams, 2006). These trials evaluate the animal’s memory of the platform location by measuring the time spent in the target quadrant and proximity to the former platform site. Administer an amnestic agent to the test grouped animals 30–60 min before the probe trial, and the control group receives only vehicle. Compare the memory performance between the test and control group (Morris, 1984).

The MWM is the most preferred and widely accepted behavioral assay and has become the heart of preclinical AD research. Multiple studies utilized the MWM to evaluate impairments in spatial learning and memory in animal model of AD. For instance, in a study by Curdt et al. (2022) researchers utilized the MWM to evaluate spatial cognition in TG4-42 mice, a transgenic AD model, at different ages (3, 7, and 12 months). While traditional performance metrics revealed noticeable memory impairments in older mice, a closer look at swimming behavior uncovered early signs of impaired spatial navigation as early as 3 months of age. These younger mice showed a preference for less efficient navigation strategies, suggesting early disruptions in spatial memory even before overt symptoms appeared (Curdt et al., 2022). Further, another study evaluated spatial learning and memory using the MWM in 5xFAD mice, considering differences related to sex and age. Both male and female mice were tested at 3, 7, and 12 months. The results showed that females developed cognitive impairments earlier, around 7 months, whereas males exhibited similar deficits later, at 12 months. These findings highlight how sex and age affect the progression of spatial memory loss in this AD model, and demonstrate the value of the MWM in detecting early-stage cognitive changes in AD models (Sánchez et al., 2023).

Despite its widespread use, the MWM presents several limitations that should be carefully considered in data interpretation. The test’s reliance on swimming ability means that physical impairments or age-related motor deficits can influence performance independently of cognitive function (Vorhees and Williams, 2006). Additionally, the stress induced by forced swimming may affect animal behavior and outcomes (D’Hooge and De Deyn, 2001). The MWM primarily assesses hippocampus-dependent spatial memory, providing limited information on other cognitive domains such as attention or executive function (Garthe and Kempermann, 2013). Furthermore, animals may adopt non-spatial strategies during the task, which can complicate the interpretation of results (Vorhees and Williams, 2006). Repeated trials may also lead to fatigue or decreased motivation, potentially impacting learning curves. While the MWM remains a valuable tool in preclinical research, its translational relevance to human cognitive process is indirect, highlighting the importance of using complementary behavioral tests to achieve a more comprehensive assessment of cognitive function in disease model (Garthe and Kempermann, 2013; Vorhees and Williams, 2006).

### 2.2 *In vitro* models

#### 2.2.1 Cellular models

##### 2.2.1.1 Immortalized cell lines and induced pluripotent stem cells (iPSCs)

Cell lines derived from human, have become a potential *in vitro* approach in the research field of AD to get deeper insights into the disease’s molecular mechanisms, and screen potential therapeutic agents. The most widely used cell lines include SH-SY5Y, H4, Neuro2A, and iPSCs, each serving distinct purposes in modeling AD-related pathologies. The SH-SY5Y cells are human neuroblastoma cell line, can be rapidly differentiated into neuron-like cells to study the pathological features of AD (Bilginer-Kartal and Arslan-Yildiz, 2025; Kola et al., 2025). Similarly, Neuro2A cells also serves to model tau aggregation, and amyloid toxicity, while the H4 cell line, overexpressing tau protein, provides insights into tauopathies, particularly tau aggregation and neurodegeneration (Qin et al., 2024). Furthermore, the advent of iPSCs, particularly derived from AD patients, has revolutionized the field, offering a personalized approach to study genetic mutations and patient-specific disease mechanisms. These cells have the ability to develop into multiple neural cell types-such as neurons, microglia, and astrocytes. Before iPSCs can be used to model AD, they must first be differentiated into relevant neural cell types to accurately replicate disease-specific cellular features (Sun et al., 2025). A 2025 study developed a 3D SH-SY5Y cell-based AD model by introducing neuronal differentiation with retinoic acid and BDNF, followed by Aβ_1–42_ exposure. The model showed distinct AD like features, and was used to test the neuroprotective potential of curcumin (Bilginer-Kartal and Arslan-Yildiz, 2025). Kola et al. (2025) utilized a nuclear magnetic resonance-based metabolomic profiling on differentiated SH-SY5Y cells to investigate the effects of natural alkaloids against Aβ-induced neurotoxicity. The findings offered valuable insights into the underlying neuroprotective mechanisms and further emphasized the relevance of this cell line as an effective *in vitro* model for AD (Kola et al., 2025). Similarly, in a 2024 study, investigators explored the effect of deubiquitinating enzyme SUP8 on AD, using H4 neuroglioma cells. They found that silencing USP8 resulted in reduced levels of β-site APP cleaving enzyme 1 (BACE1) and Aβ, suggesting the potential of this cell line for modeling AD (Qin et al., 2024). Furthermore, Sun et al. (2025) developed a novel tauopathy model with four-repeat (4R) tau protein using Clustered Regularly Interspaced Short Palindromic Repeats (CRISPR)-edited iPSC-derived neurons. By utilizing CRISPR interference screening, they identified key genetic factors involved in the propagation of tau, shedding light on the molecular mechanisms underlying tau-related neurodegeneration (Sun et al., 2025).

While these cell lines offer significant advantages, including genetic consistency, human relevance, and cos-effective platform for drug screening, they also possess notable limitations. The absence of complex interactions between different cell types in a whole organism and the non-physiological conditions of *in vitro* environments may hinder the full recapitulation of AD pathology. Despite these challenges, human neuronal cell lines remain essential in advancing our understanding of AD and in identifying potential therapeutic strategies (Bilginer-Kartal and Arslan-Yildiz, 2025; Kola et al., 2025; Qin et al., 2024; Sun et al., 2025).

##### 2.2.1.2 Primary neuronal culture

Primary neuronal cultures, commonly derived from hippocampus or cortex of embryonic or neonatal rodents, serves as a valuable *in vitro* system for studying various neurodegenerative diseases including AD. These cultures maintain key structural and functional characteristics of neurons observed *in vivo* including neurotransmitter activity, synaptic connectivity, and electrophysiological behavior. Due to their relevance, they are frequently used to explore cellular and molecular processes linked to AD pathogenesis. Specifically, they provide robust platform to investigate the effects of tau-related dysfunction, Aβ accumulation, and downstream neurodegenerative mechanisms under controlled laboratory conditions (Li et al., 2023; Sharma et al., 2021).

The significance of primary neuronal cultures in AD research stems from their sensitivity to disease-relevant insults. When treated with synthetic Aβ oligomers, these neurons replicate several key features of AD pathology. Likewise, exposure to tau proteins or genetic overexpression of tau mimics tauopathy-associated neuronal damage, such as destabilization and hyperphosphorylation of microtubules. These models enable detailed monitoring of neuronal responses through techniques like immunocytochemistry, live-cell imaging, electrophysiological recordings, calcium flux assays, and molecular profiling using tools such as qPCR and wester blotting (Sharma et al., 2021; Teklemariam et al., 2025). A study by Li et al. (2023) employed primary neuronal cultures to examine the effects of tau overexpression. The results demonstrated that wild-type human tau triggered DNA damage and activated MAPK-DLK signaling, leading to neuronal degeneration. Interestingly, the P301L tau variant caused DNA damage without marked neurodegeneration. These findings emphasize the sensitivity of primary neurons to tau-induced stress, underlining their value in modeling AD related mechanisms (Li et al., 2023).

##### 2.2.1.3 Co-culture (neuronal-glial) model

The neuronal-glial co-culture model has been gaining extreme attention in AD research. This model provides greater insights into role of neuroinflammation, and synaptic dysfunction in pathogenesis of AD. In AD, glial cells-especially microglia, are central to neuroinflammation that can intensify neuronal injury. Conversely, astrocytes play a protective role by supporting synaptic integrity and maintain the blood brain barrier (Kim et al., 2024; Supakul et al., 2024). By culturing neurons alongside glial cells, co-culture models enable the study of how these cellular interactions influence the development and progression of AD. This approach provides valuable insights into the cellular and molecular mechanisms that drive the disease. Preclinical studies have been extensively utilizing this model for screening various therapeutic agents as well as potential therapeutic targets (Kim et al., 2024; Luchena et al., 2022). For example, research employed a tri-culture model integrating primary neurons, astrocytes, and microglia to explore how these cells collectively respond to Aβ exposure. The results emphasized that interactions between neurons and glial cells significantly influenced microglial activity, which in turn modulated inflammatory responses and contributed to neurodegeneration (Kim et al., 2024). Similarly, another investigation developed a 2D co-culture system incorporating neurons, microglia, and astrocytes to model AD. Following treatment with oligomeric Aβ, the culture exhibited hallmark AD features such as excessive microglial activation, and synaptic degradation (Luchena et al., 2022).

As this model offers a more physiologically relevant system to investigate AD pathology and potential therapeutic targets, it may serve as an ideal alternative to animal studies. However further research is required for more comprehensive inclusion of this approach in AD research.

#### 2.2.2 Three dimensional (3D) and organoid model

Three-dimensional (3D) and brain organoid models offer a more physiologically relevant alternative to conventional two-dimensional (2D) cultures for studying AD and other neurological conditions. These systems are typically developed from iPSCs or neural progenitor cells, which self-organize into structured, multilayered aggregates resembling early brain architecture (Choi et al., 2014; Lancaster and Knoblich, 2014). This spatial organization supports more realistic cellular interaction, neuronal network formation, and neurotransmission (Raja et al., 2016). Compared to 2D models, 3D systems better replicate disease-associated changes, including tau pathology, amyloid-beta accumulation, and in some cases, early signs of neuroinflammation, particularly when glial cells are incorporated. Their ability to sustain long-term cultures enables researchers to monitor progressive neurodegeneration and assess chronic drug effects (Park et al., 2018). A wide range of studies had utilized this model to dissect molecular mechanisms underlying AD, given its strong pathological similarity to the human condition. For example, Choe et al. (2024) developed a familial AD model using cerebral organoids derived from genetically modified human pluripotent stem cells. These organoids exhibited AD related changes, such as tau alterations, amyloid accumulation, and neuronal loss. Notably, treatment with secretase inhibitors reduced these pathological features, highlighting the model’s value for investigating disease mechanisms and therapeutic strategies (Choe et al., 2024). Further another investigation employed cerebral organoids integrated with microglia from human pluripotent stem cells to explore neuroinflammatory processes in AD. The model revealed distinct and evolving microglial activation states, some of which were linked to key pathological features of AD (Kuhn et al., 2023). Additionally, Lavekar et al. (2023) developed 3D retinal organoids using human stem cells with familial AD mutations. These organoids demonstrated elevated Aβ 42:40 ratios and increased tau phosphorylation, reflecting essential AD characteristics. Changes in gene expression related to synaptic functions were also observed, supporting the use of retinal organoids to investigate early AD mechanisms (Lavekar et al., 2023).

Although, 3D and organoid platforms represent a significant advancement in the *in vitro* modeling of AD, offering valuable insights into complex disease mechanisms and treatment responses, several challenges such as variability in organoid formation and the lack of full vascularization still limit their full potential (Kim et al., 2015). Therefore, additional studies are needed to overcome these limitations by enhancing the development, consistency, and blood vessel formation in organoid models, which will increase their effectiveness for AD research and drug development.

### 2.3 *In vivo* models

*In vivo* models are vital for studying AD, as they replicate key aspects of its pathology and support the development and testing of potential therapies. Commonly used models include genetically modified rodents, drug-induced models, and aged animals, each helping researchers explore disease mechanisms and evaluate treatment outcomes in a biologically relevant context (Dhapola et al., 2023).

This section emphasizes on some commonly used *in vivo* models in AD research.

#### 2.3.1 Transgenic model

Transgenic models play a pivotal role in advancing our understanding of AD. These genetically engineered models are developed to recapitulate key pathological hallmarks of AD, such as Aβ accumulation, neuronal loss, and cognitive decline (Pádua et al., 2024). Various transgenic strains (see Table 2), including APP, APP/PS1, 3xTg-AD, have been extensively used to explore different aspects of AD pathophysiology. These models enable the investigation of genetic risk factors, elucidation of disease mechanisms, and evaluation of the effects of AD on systemic functions. Furthermore, they serve as valuable tools for testing potential therapeutic interventions and understanding the hereditary nature of the disease (Kitazawa et al., 2012; Pádua et al., 2024).

**TABLE 2:** Currently available genetic models for AD.

Model	Genetic alterations	Main features	Application in AD research	References
APP/PS1	Human APP with Swedish mutation and PSEN1 ΔE9	Develops amyloid plaques and glial activation with moderate cognitive decline	Useful for studying amyloid-targeted therapies	Wang et al., 2024; Xu et al., 2024
3xTg-AD	APP (Swedish), PSEN1 (M146V), Tau (P301L)	Shows both amyloid and tau pathology, synaptic damage, and memory impairments	Suitable for evaluating multitarget strategies involving Aβ and tau	Oddo et al., 2003
5xFAD	APP (3 mutations), PSEN1 (2 mutations)	Strong inflammatory response, rapid plaque formation, early cognitive changes	Suitable for screening fast-acting therapeutic agent targeting amyloidosis	Forner et al., 2021
Tau P301S	MAPT gene carrying P301S mutation	Demonstrates tangle pathology, neuronal loss, and motor	Used in tauopathy-focused interventions and mechanistic research	Yoshiyama et al., 2007
Tg2576	Human APP with Swedish mutation	Produces Aβ deposits and exhibits deficits in learning and synaptic function	Ideal for studying progressive stages of amyloid accumulation	Hsiao et al., 1996

##### 2.3.1.1 APP transgenic mice model

Amyloid precursor protein (APP) transgenic mice are among the most widely employed animal models in AD research. These mice are genetically engineered to overexpress human APP, often incorporating mutations associated with familial AD. This modification leads to the excessive production of Aβ peptides, which aggregate to form plaques-one of the defining pathological features of AD. The accumulation of Aβ plaques in these models mimics the neurodegenerative process seen in human AD, making them essential for investigating disease mechanisms and testing potential therapeutic strategies aimed at modulating Aβ-related pathophysiology. Importantly, the overexpression of APP influences multiple neuronal populations, particularly the cholinergic neurons located in the basal forebrain. The degeneration of these neurons leads to disrupted signaling toward key cognitive regions such as the hippocampus and neocortex, which is associated with deficits in attention and memory. These neuronal impairments are reflected in the cognitive and behavioral abnormalities observed in APP transgenic mouse models (Lee and Chen, 2024; Perez et al., 2007). This model has demonstrated robust and informative outcomes across various preclinical studies. For instance, Blackmer-Raynolds et al. (2025) followed APP knock-in (APP*^SAA^*) mice for 16 months to observe changes in behavior. Both APP*^SAA^* and wild-type mice showed motor impairments with age, but only APP*^SAA^* mice had memory loss (Blackmer-Raynolds et al., 2025). Further, another study using APP*^SWE^*/PS1ΔE9 transgenic mice, showed that these animals developed early and progressively worsening damage to cholinergic fibers in the hippocampus and cortex. This degeneration coincided with the accumulation of Aβ, suggesting that APP overexpression compromised the integrity of cholinergic projections in these brain regions (Perez et al., 2007). These findings emphasize the relevance of APP transgenic mice as a valuable tool for exploring AD mechanisms and for preclinical assessment of potential treatments.

##### 2.3.1.2 APP/PS1 transgenic model

Another commonly used mouse model in AD research is the APP/PS1 transgenic model. The model combines mutations in both APP and Presenilin (PS1) genes. This dual transgenic approach enables the study of Aβ deposition, neuroinflammation, and cognitive decline- key pathological features of AD. In this model, the APP mutation typically involves the Swedish mutation (K670N/M671L), leading to increased production of Aβ peptides, particularly Aβ42, while PS1 mutation (such as PS1 M146L) enhances γ-secretase activity, promoting Aβ production. Numerous preclinical evidences provided substantial evidence of how APP and PS1 mutation results in cognitive impairment, with the onset of Aβ plaque deposition (Lok et al., 2013; Zhu et al., 2017). Within this experimental framework, the buildup of pathological Aβ is found to impact specific neuronal populations, including pyramidal neurons in the hippocampus and cholinergic neurons in the basal forebrain. The decline in cholinergic signaling to the hippocampus and cortex is associated with impairments in spatial learning and memory functions (Gengler et al., 2010; Li et al., 2013). In fact a MRI study revealed various brain changes in APP/PS1 transgenic mice, such as disruptions in white matter, atrophy of the hippocampus, and decreased functional connectivity. These alterations were detected before the onset of notable cognitive impairments, which were observed in the later stages of the disease (Xu et al., 2024). Further, Wang et al. (2024) conducted a label-free quantitative proteomic analysis to investigate hippocampal protein alterations in APP/PS1 transgenic mice, a model of AD. Over the course of aging from 2–12 months, 396 out of 2762 quantifiable proteins showed significant changes, indicating disruptions linked to amyloid pathology. Among these, 35 proteins demonstrated consistent expression shifts with age, mirroring the gradual progression of amyloid accumulation and neuronal damage. These findings highlight the role of APP and PS1 mutations in driving age-dependent proteomic changes, contributing to early disease mechanisms and identifying possible therapeutic targets for AD (Wang et al., 2024).

Although the APP/PS1 transgenic model has significantly contributed in advancing AD research, it presents several important limitations. A major drawback is the absence of tau pathology, such as neurofibrillary tangles and associated neurodegeneration in AD (Jankowsky and Zheng, 2017). This restricts the model’s capacity to fully replicate the complex neurofibrillary aspects of the disease. Furthermore, the overexpression of mutant APP and PS1 genes- beyond physiological levels-may lead to artifacts that don’t accurately reflect the natural course of AD (Sasaguri et al., 2017). Such supraphysiological expression can potentially affect the reliability of therapeutic outcomes and limit the translational validity of preclinical findings (Onos et al., 2019). Consequently, while the APP/PS1 model is highly effective for studying amyloid-related pathology, it may not fully capture the multifunctional nature of sporadic AD (Saito et al., 2014).

##### 2.3.1.3 3xTg-AD mice model

It is a widely utilized transgenic model in AD research due to its incorporation of three human mutant genes associated with AD pathology: APP, PS1, and tau. This model expresses the APP Swedish mutation (K670N/M671L), the PS1 M146V mutation, and the tau P301L mutation-each contributing to the development of AD features such as Aβ plaques, tau hyperphosphorylation, and neurofibrillary tangle formation (Oddo et al., 2003). These genes are typically driven by neuron-specific promoters to ensure expression within relevant brain regions. The generation of the model involves microinjection of the transgene constructs into fertilized mouse embryos at the pronuclear stage, followed by implantation into pseudo-pregnant female mice (Gordon and Ruddle, 1981). Investigators extensively included this model in numerous AD studies, owing to its ability to reliably replicate key neuropathological features of the disorder. For example, Judd et al. (2024) utilized a Intellicage system to investigate cognitive alterations in 11.5 month-old 3xTg-AD mice. The transgenic mice exhibited a marked increase in premature responses and a reduction in correct responses during reaction time tasks, indicative of heightened impulsivity and attention deficits. These impairments were significantly associated with elevated cortical Aβ and phosphorylated tau levels, underscoring early functional consequences of AD-related neuropathology (Judd et al., 2024). In addition, this model has been instrumental in exploring sex-specific variations in disease progression. One investigation revealed distinct neuroinflammatory profiles and differential pathology between male and female mice, highlighting the importance of sex as a biological variable in AD research (Barber et al., 2024). Similarly another study included the same model to explore the effects of repetitive transcranial magnetic stimulation on behavioral alteration, highlighting its broader applicability in the research field (Kraybill et al., 2024).

As the 3xTg-AD model exhibits both amyloid and tau pathology, it provides a more comprehensive representation of AD pathogenesis compared to single-transgene models. Overall, this model stands out among transgenic models for its capacity to mirror multiple aspects of human AD pathology, making it particularly valuable for preclinical studies focused on disease mechanisms and therapeutic development.

#### 2.3.2 Non-transgenic (induced) model

Induced models of AD are established by administering chemical, biological, or physical agents (described in Tables 3, 4) to replicate core pathological hallmarks of the disease. Unlike transgenic models, these approaches don’t involve genetic alterations, making them especially relevant for investigating sporadic AD, which represents the predominant form observed in humans.

**TABLE 3:** Commonly used chemical agents to induce AD.

Agents	Mechanism	Effects	Models	References
STZ	Impaired Insulin signaling, oxidative stress and mitochondrial dysfunction, cholinergic dysfunction, neuroinflammation	Lowered glucose uptake and metabolism in neurons, Energy insufficiency and neuronal apoptosis, neuronal degeneration	*i.c.v.* injection in rodents	Chen et al., 2013
Aβ (Aβ1-42, Aβ1-41) oligomers	Tau hyperphosphorylation, membrane disruption and calcium dysregulation, synaptic dysfunction	Disruption of calcium homeostasis and neuronal toxicity, damages cellular components, including lipids, proteins, and DNA. Neurofibrillary tangles and neuronal degeneration.	Intra-hippocampal and intracranial injection	Alkandari et al., 2023
AlCl_3_	Oxidative stress and mitochondrial dysfunction, glial activation and neuroinflammation, tau hyperphosphorylation	ATP depletion and neuronal apoptosis, neurofibrillary tangles, synaptic dysfunction and cognitive impairment	Intraperitoneal, *i.c.v.* injection to rodents	Chen et al., 2021
Okadaic acid	Inhibits protein phosphatases 1 and 2A, induces oxidative stress	Excessive tau phosphorylation, tau aggregation and neurofibrillary tangle formation, mitochondrial dysfunction and neurodegeneration	Hippocampal injection	Kamat et al., 2023
Lipopolysaccharide	Toll-like receptor 4 and activate nuclear factor-kappa B signaling	Release of pro-inflammatory cytokines such as TNF-α, IL-1β, and IL-6. Neuroinflammation	Systemic or intracerebral injection	Zhao et al., 2017

**TABLE 4:** Heavy metals used to induce AD.

Metals	Mechanism	Effects	Models	References
Aluminum	Promotes reactive oxygen species (ROS), tau hyperphosphorylation, disrupt kinase and phosphatase activity	Aβ Aggregation and plaque formation, neurofibrillary tangles, disrupt iron homeostasis	Long term exposure	Gangarde et al., 2025
Mercury	Oxidative stress, DNA damage and epigenetic modifications	Neuronal degeneration, altered gene expression	Chronic exposure	Zahed et al., 2024
Lead	Activates microglia and astrocytes, upregulates β-secretase and downregulates α-secretase, ROS generation, alters DNA methylation and histone modifications	Promoting Aβ production and reducing its clearance, aggregation of Aβ plaques, dysregulated gene expression	Chronic exposure via Inhalation and ingestion	Qiao et al., 2024
Copper	Copper binds to Aβ peptides, enhancing their aggregation into toxic oligomers and fibrils, The Cu-Aβ complex enhances ROS generation	Promotes oxidative stress, neuronal toxicity, neurofibrillary tangles	Long term dietary exposure	Huang et al., 2024

This section emphasizes on few of these non-transgenic approaches.

##### 2.3.2.1 Aβ induced model

This widely used model is a non-transgenic model, mimicking early pathological features of AD. It is developed by delivering the synthetic Aβ peptides (especially Aβ_1–42_ due to its high tendency for aggregation and neurotoxicity) through intra cerebroventricular (*i.c.v.*) or direct intra-hippocampal injection (Cormio et al., 2005; Pritchard et al., 2008). After administration, the peptides tend to aggregate into oligomers and fibrils, triggering a cascade of pathological processes, including synaptic impairment, oxidative damage, neuroinflammation, and ultimately neuronal cell death. The model has been exclusively employed in numerous preclinical studies as it provides broader aspects of AD pathology (Facchinetti et al., 2018; Pritchard et al., 2008). For instance, *i.c.v.* administration of Aβ_1–40_ in rats showed impair spatial memory and induce systemic inflammation, underscoring the contribution of peripheral immune responses to AD pathology (Nefodova et al., 2024).

In another research, a mouse model was developed by bilaterally injecting Aβ_1<suprm>–</suprm>42_ oligomers into the hippocampal CA1 regions, resulting cognitive impairment. The model was subsequently used to evaluate the therapeutic potential of cajaninstilbene acid (Wang et al., 2019). Moreover, oral supplementation with *Lactobacillus gasseri* and *Lacticaseibacillus rhamnosus* improved cognitive function in Aβ-induced mouse models (Choi et al., 2025).

These findings highlight the value of Aβ-induced animal models in elucidating the mechanisms driving cognitive decline in AD and in assessing the efficacy of potential therapeutic strategies.

However, these models have certain limitations, such as the absence of tau-related pathology, minimal progressive neurodegeneration, and significant variability influenced by differences in peptide preparation and administration methods (Collins and Greenfield, 2024).

##### 2.3.2.2 STZ induced model

The STZ-induced model is a well-established approach used to mimic the sporadic AD, which constitutes the majority of clinical cases. The model is developed by *i.c.v.* administration of STZ, a compound that impairs insulin signaling within the brain. This insulin resistance results in reduced glucose metabolism, mitochondrial dysfunction, oxidative stress, and ultimately cognitive decline (Biswas et al., 2016). Apart from cognitive decline this model also exhibits cholinergic dysfunction, elevated Aβ levels, and tau hyperphosphorylation, making it suitable for studying amyloid and tau pathways (Guimarães-Souza et al., 2011). For example, a 2025 study employed STZ-induced model to demonstrate the neuroprotective role of neuritin against cognitive deficits and synaptic dysfunction (Kazemi et al., 2025). Another investigation revealed that overexpression of peroxiredoxin 1 effectively reduced oxidative damage and neuronal loss in STZ-treated rats, suggesting its antioxidant-based neuroprotection (Park et al., 2024). Interestingly, combination of peripheral STZ administration with high-fat diet was reported to exacerbate tau hyperphosphorylation, Aβ accumulation, and pronounced cognitive deficits-thereby providing a more comprehensive model of sporadic AD (Sun et al., 2024).

##### 2.3.2.3 Scopolamine-induced amnesia

Scopolamine is muscarinic acetylcholine receptor antagonist. It impairs cholinergic neurotransmission- a pathway that is critically involved in learning and memory processes, and significantly disrupted in AD. By blocking central muscarinic receptors, scopolamine administration leads to transient cognitive impairments, especially in working memory, attention, and spatial learning. Scopolamine has been extensively utilized in both clinical and preclinical studies to induce transient cognitive deficits, particularly affecting learning and memory, thereby serving as a reliable pharmacological model for assessing the efficacy of cognition-enhancing agents. Recent studies have provided insights into the underlying mechanisms of cognitive impairment following scopolamine exposure. A 2023 study reported that scopolamine administration led to disrupted mitochondrial dynamics and synaptic plasticity in the hippocampus, contributing to memory deficits (Mishra and Thakur, 2023). Another investigation showed that barbigerone, a natural antioxidant compound, significantly reversed scopolamine-induced cognitive dysfunction by reducing oxidative stress and acetylcholinesterase activity, along with modulating neuroinflammatory pathways (AlGhamdi et al., 2023). In addition, McGugin et al. (2023) demonstrated that scopolamine caused functional impairments in brain network activity resembling delirium, which were reversible and occurred without permanent neuronal damage, supporting the model’s applicability for studying transient cognitive decline (McGugin et al., 2023).

These findings collectively highlight the utility of the scopolamine model in exploring mechanisms of cholinergic-associated cognitive decline and evaluating potential neuroprotective agents in AD research.

##### 2.3.2.4 Aluminum chloride (AlCl_3_) induced model

It is a well-recognized approach to stimulate AD pathology in rodents. Chronic exposure to AlCl_3_ leads to cholinergic dysfunction, oxidative stress, neuroinflammation, and ultimately cognitive impairments, mirroring the key aspects of AD. Recent studies have widely explored this model to screen various therapeutic agents to counteract these effects. For instance, Ononin, a bioactive isoflavone, was found to improve memory performance and modulate oxidative and inflammatory markers in AlCl_3_-exposed rats, demonstrating its therapeutic potential in neurodegeneration (Chen et al., 2021). Similarly, nattokinase, an enzyme derived from fermented soy, showed significant cognitive benefits in rats co-administered with AlCl_3_ and D-galactose by reducing amyloid pathology and brain atrophy (Tanikawa et al., 2024). Additionally, a combination treatment with baicalein and memantine has also shown enhanced neuroprotection in AlCl_3_-exposed animals (Jadhav and Kulkarni, 2023). Overall, this model remains a relevant and versatile tool for investigating the mechanisms of cognitive decline and screening potential interventions for AD.

While non-transgenic or induced models are widely used in AD research due to their simplicity, low cost, and ability to quickly induce cognitive deficits, they don’t accurately mimic the full pathological progression seen in human AD. Most of these models, including those induced by phenytoin, scopolamine, STZ, or AlCl_3_, can replicate behavioral symptoms such as memory loss, but they fall short in representing core histopathological features such as Aβ deposition, neurofibrillary tangle formation, and widespread synaptic and neuronal degeneration (Javed and Ojha, 2022; Müller et al., 2021). The cognitive impairments observed in these models are generally attributed to secondary mechanisms like oxidative damage, neuroinflammation, cholinergic dysfunction, or metabolic stress rather than direct Aβ or tau pathology. As such, while they are useful for screening neuroprotective agents or studying specific biochemical pathways, they offer limited insight into the progressive and multifactorial nature of AD. Moreover, these models lack genetic alterations that are associated with familial AD, such as mutations in APP, PSEN1, or MAPT (Auxtero et al., 2021; Bjorkli et al., 2020; Calderón-Garcidueñas and de la Monte, 2017). This limits their relevance for studying the genetic underpinnings of the disease or for evaluating therapies aimed at disease-modifying targets like amyloid and tau. Therefore, results obtained from these models should be interpreted with caution, particularly when extrapolating to clinical scenarios. To increase the translational significance of pharmacologically induced models, it is advisable to pair behavioral assessments with molecular and histological validations, or to use them in conjunction with transgenic or humanized models. This complementary approach can help clarify the relationship between observed cognitive deficits and the underlying disease mechanisms, ultimately strengthening the preclinical evaluation of candidate therapies.

#### 2.3.3 Natural and accelerated aging model

The natural aging models serves as a valuable tool in AD study, as it closely reflects the gradual cognitive and neurobiological decline seen in human aging. Aged rodents, particularly rats and mice, are frequently used due to their short lifespan, genetic tractability, and well-understood physiology. Additionally, non-human primates such as marmosets and rhesus macaques offer an advanced model due to their complex brain structures and closer phylogenetic relation to humans (Arnsten et al., 2019; Li and Gu, 2023).

Animals are typically housed in standard laboratory conditions and divided into age-specific groups to examine age-dependents changes. Behavioral assessments like the fear conditioning, MWM, and novel object recognition are commonly employed to evaluate learning and memory performance (Burke et al., 2019; Gallagher and Rapp, 1997). These tests help detect subtle cognitive impairments that emerge with age. Further, neuropathological evaluations, including histological analysis of post-mortem brain tissue, reveal key AD hallmarks such as amyloid plaques, neurofibrillary tangles, neuroinflammation, and synaptic loss. Additionally, biochemical techniques like mass spectrometry and enzyme linked immunosorbent assay can be used to quantify AD-related biomarkers such as Aβ peptides and phosphorylated tau. This model allows for longitudinal studies, offering insights into the natural trajectory of brain aging and its role in AD development (Fisher and Hanin, 1986; Pepeu, 2001).

In contrast to natural aging models, accelerated aging models employ genetic, chemical, or environmental methods to speed up the aging process and trigger early development of AD-like symptoms within a shorter period. This strategy enables researcher to investigate the underlying biological processes of AD and test new treatments more rapidly compared to traditional natural aging models. Earlier researches had employed this model for shorter evaluation periods to complete studies on the efficacy of AD therapies. For instance, a 2024 study showed that oral treatment with Formononetin improved memory, reduced neuroinflammation, and restored antioxidant signaling in SAMP8 mice model of AD. Behavioral tests, brain histology, and transcriptomic analysis confirmed that Formononetin protected neurons and modulated key genes linked to oxidative stress and inflammation, suggesting its protentional as a therapeutic agent for age-related cognitive decline (Liu et al., 2024). Further, another study explored the impact of Diminazene Aceturate, an activator of angiotensin converting enzyme-2, on AD-like brain pathology and cognitive deficits using SAMP8 mice. Results showed that treatment group increased levels of angiotensin-(1-7) and its receptor MAS1 in the brain, while lowering Aβ_142_ accumulation and tau protein hyperphosphorylation. Additionally, the treatment reduced cytokine expression and helped preserve synaptic and neuronal integrity. Behavioral tests, including the MWM, demonstrated that the treatment enhanced spatial learning and memory, underscoring its potential as a therapeutic approach and the usefulness of the SAMP8 model for studying accelerated aging in AD research(Duan et al., 2020).

Although, aged animals may not develop robust amyloid plaques or neurofibrillary tangles comparable to those seen in transgenic models, they are valuable for evaluating interventions aimed at delaying or preventing age-related cognitive decline.

#### 2.3.4 Emerging approaches

Non-mammalian models, such as *Danio rerio* (zebrafish) and *Drosophila melanogaster* (fruit fly) have emerged as valuable tools in AD research due to their genetic tractability, cost-effectiveness, and short life cycles. These models allow for rapid screening of genetic and pharmacological interventions and enable the study evolutionarily conserved molecular pathways involved in neurodegeneration (Chia et al., 2022).

##### 2.3.4.1 *Drosophila melanogaster* model of AD

Fruit fly has emerged as a powerful and genetically tractable model for studying the pathophysiology of AD. Its relatively simple nervous system, highly conserved genetic pathways, and short life cycle allow for efficient modeling of neurodegenerative disorders. Transgenic flies expressing human Aβ peptides or mutant tau proteins under tissues-specific promoters, develop progressive neurodegeneration, memory impairments, locomotors deficits, and reduced lifespan-phenotypes that mirror key aspects of AD (Bongiorni et al., 2024; Elovsson et al., 2024; Yang et al., 2024). These models have proven instrumental in dissecting the molecular mechanisms of Aβ toxicity, including protein aggregation, and synaptic dysfunction. For instance, Yang et al. (2024) employed a *Drosophila* model to investigate the effect of Nesfatin-1 on tau pathology. The results revealed that Nesfatin-1 overexpression in flies expressing human tau protein, reduced the levels of human tau protein and mitigated neurodegenerative phenotypes (Yang et al., 2024). In addition, investigators developed a transgenic *Drosophila* AD model to explore the therapeutic potential of Lisosan G. They demonstrated that Lisosan G ameliorated the tau-induced neurodegenerative symptoms (Bongiorni et al., 2024). Further, researchers developed a new *Drosophila* model of AD by expressing Aβ in the fly’s intestine. This model exhibited toxic effects similar to previous neuronal Aβ-expressing fly models and can be used to advance our understanding of the mechanism of Aβ proteotoxicity (Elovsson et al., 2024).

##### 2.3.4.2 *Danio rerio* (zebrafish) model

The Zebrafish has emerged as a valuable model in AD research due to its genetic homology with humans, transparent embryos, and compatibility with high-throughput screening. They possess orthologs of key human AD-related genes such as APP, PSEN _(1<suprm>–</suprm>2)_, and MAPT, making the ideal for modeling the molecular underpinnings of AD. This model can be developed through genetic mutations, and physical or chemical induction. These models successfully mimic the AD pathology; thus, recent investigations have widely employed these models in their studies. A transcriptomic study of 7 day-old Zebrafish larvae with a PSEN1 mutation, resembling familial AD, identifies disruptions in oxidative phosphorylation, mini-chromosome maintenance, extracellular matrix remodeling, and iron homeostasis. These findings suggest that this Zebrafish model offers valuable insights into the molecular mechanisms underlying AD (Dong et al., 2021). Valu et al. (2021) developed an AD model in Zebrafish using scopolamine to investigate the neuroprotective effect of an ethanolic extract from *Hericium erinaceus* (Lion’s Mane mushroom). The results revealed that the administration of the extract significantly improved the memory performance, further highlighting the potential applicability of this model in the field of AD research (Valu et al., 2021).

Although, this model is still in its very early stage, in depth research is required to fully explore this model at advanced molecular level.

## 3 Translational challenges of rodent models in representing early-stage AD

Rodent models have significantly contributed to the study of AD, particularly in uncovering molecular mechanisms such as tau alterations, Aβ deposition, and neuroinflammatory processes (Ashe and Zahs, 2010; LaFerla and Green, 2012). However, they present notable limitations when it comes to reflecting the early, preclinical or prodromal stages of AD as seen in humans. A majority of these models are genetically engineered to express mutations associated with familial AD-mutations in genes like APP, PSEN1, or PSEN2-which are rare in the broader population (Jankowsky and Zheng, 2017). Consequently, these models often display early-onset Aβ pathology but don’t adequately represent the sporadic, age-associated nature of most AD cases (Sasaguri et al., 2017). In addition, critical aspects such as age-related physiological decline, metabolic disturbances, and vascular contributions are either absent or not well replicated in rodents (Drummond and Wisniewski, 2017). Structural and functional differences between rodent and human brains, such as variation in cortical organization and cognitive capabilities, further hinder the modeling of complex, gradual symptom progression seen in human AD. Notably, many rodent models don’t naturally develop tau pathology or exhibit substantial neuronal degeneration and brain atrophy-key features of advanced human AD (Do Carmo and Cuello, 2013; Onos et al., 2016). These gaps contribute to a lack of predictive validity, as many therapeutic strategies that show efficacy in animal studies fail during clinical trials (Cummings et al., 2014). Therefore, while rodent models remain useful for exploring disease mechanisms, their ability to model the initial stages of AD remains limited. To improve translational success, there is a growing emphasis on integrating alternative models, including iPSC-derived human neuronal cultures and higher-order species like non-human primates, which may better recapitulate early disease biology (Choi et al., 2014; Izpisua Belmonte et al., 2015).

## 4 Future perspectives

The ongoing evolutions of screening models for AD continues to be essential for advancing our understanding of disease mechanisms and therapeutic development. Although current models-ranging from pharmacological to transgenic animal model- have provided valuable insights, they often fall short in fully replicating the complex and multifactorial nature of human AD pathology (Dawson et al., 2018). As a result, future efforts must focus on developing models with enhanced translational relevance and predictive power. Emerging approaches such as the use of iPSCs derived from AD patients offer promising avenues for personalized modeling. These models can mimic patient-specific phenotypes, enabling a better understanding of disease heterogeneity and individualized drug response (Shi et al., 2012; Sun et al., 2025). Furthermore, the advancement of 3D brain organoids and microfluidic organ-on-chip systems represents a significant step toward, as they recreate a more physiologically relevant neural environment, including multicellular interactions and structural complexity (Choi et al., 2014). Additionally, the integration of high-throughput omics technologies-such as proteomics, transcriptomics, and metabolomics-into experimental workflow is expected to facilitate the identification of novel biomarkers and therapeutic targets. Genetic engineering tools like CRISPR/Cas9 also hold potential for generating refined animal models that closely mimic human AD mutations, aiding in the study of both familial and sporadic form of the disease (Israel et al., 2012; Nativio et al., 2018; Proville et al., 2014; Sun et al., 2025). Moreover, the application of artificial intelligence and machine learning to preclinical research can optimize model selection, accelerate data interpretation, and predict treatment outcomes more effectively. By combining *in vitro* and *in vivo* models with computational tools, researchers can construct comprehensive disease frameworks that more accurately mirror human AD progression. Overall, a multidisciplinary approach combining cellular, molecular, computational, and behavioral sciences will be essential to develop more predictive and reliable screening models. Such advancement will not only deepen our understanding of AD pathogenesis but also accelerate the discovery of effective therapeutic strategies.

## 5 Conclusion

The use of screening models is very crucial in deepening our understanding of AD and in search for effective therapeutic strategies. A diverse range of *in vivo* models-including pharmacologically induced models, transgenic animal models, and *in vitro* neuronal cell cultures-each with distinct advantages for exploring various pathological mechanisms of AD, such as tau pathology, Aβ aggregation, synaptic dysfunction, and neuroinflammation. These models have been instrumental in identifying potential drug candidates, therapeutic targets, and disease biomarkers. By replicating key features of AD pathology, they enable the preclinical evaluation of potential treatments, ultimately facilitating the transition to clinical studies. However, given the complex and multifaceted nature of AD, it is essential to recognize that no single model can fully encompass all aspects of the human condition. Thus, results from these models should be interpreted with care, and validation across multiple model system is necessary. Ongoing advances in model refinement, including the integration of patient-specific iPSCs, multi-omic approaches, and gene editing technologies, will continue to improve the relevance and predictive power of AD models. Recent progress in AD research has highlighted the critical role of early detection, individualized treatment approaches, and the simultaneous targeting of multiple disease mechanisms. Significant strides in biomarker development-such as blood-based tests for phosphorylated tau and Aβ oligomers-have opened new possibilities for early and less invasive diagnosis. The recent regulatory approvals of anti-amyloid monoclonal antibodies, including Lecanemab and Donanemab, represent a notable shift toward disease-modifying therapies in clinical practice. In parallel, the application of artificial intelligence, integration of real-world evidence, and systems-level approaches are enhancing the efficiency and personalization of drug-discovery pipelines. As these technologies continue to evolve alongside advanced model systems, they will be essential in driving the development and clinical translation of more effective treatments for this complex and progressive neurodegenerative disease.
